# Maximum occlusal force and medial mandibular flexure in relation to vertical facial pattern: a cross-sectional study

**DOI:** 10.1186/1746-160X-3-18

**Published:** 2007-04-02

**Authors:** Rosemary S Shinkai, Fabio L Lazzari, Simone A Canabarro, Márcia Gomes, Márcio L Grossi, Luciana M Hirakata, Eduardo G Mota

**Affiliations:** 1 Department of Prosthodontics, Pontifical Catholic University of Rio Grande do Sul, Porto Alegre, RS, Brazil; 2 Graduate Program in Dentistry, Pontifical Catholic University of Rio Grande do Sul, Porto Alegre, RS, Brazil; 3 Private Practice, Caxias do Sul, RS, Brazil; 4 Graduate Program in Dentistry, Federal University of Rio Grande do Sul, Porto Alegre, RS, Brazil

## Abstract

**Background:**

Vertical facial pattern may be related to the direction of pull of the masticatory muscles, yet its effect on occlusal force and elastic deformation of the mandible still is unclear. This study tested whether the variation in vertical facial pattern is related to the variation in maximum occlusal force (MOF) and medial mandibular flexure (MMF) in 51 fully-dentate adults.

**Methods:**

Data from cephalometric analysis according to the method of Ricketts were used to divide the subjects into three groups: Dolichofacial (n = 6), Mesofacial (n = 10) and Brachyfacial (n = 35). Bilateral MOF was measured using a cross-arch force transducer placed in the first molar region. For MMF, impressions of the mandibular occlusal surface were made in rest (R) and in maximum opening (O) positions. The impressions were scanned, and reference points were selected on the occlusal surface of the contralateral first molars. MMF was calculated by subtracting the intermolar distance in O from the intermolar distance in R. Data were analysed by ANCOVA (fixed factors: facial pattern, sex; covariate: body mass index (BMI); alpha = 0.05).

**Results:**

No significant difference of MOF or MMF was found among the three facial patterns (P = 0.62 and P = 0.72, respectively). BMI was not a significant covariate for MOF or MMF (P > 0.05). Sex was a significant factor only for MOF (P = 0.007); males had higher MOF values than females.

**Conclusion:**

These results suggest that MOF and MMF did not vary as a function of vertical facial pattern in this Brazilian sample.

## Background

The relationship between vertical facial pattern and masticatory muscle anatomy and function still is controversial in the literature. It has been reported that masseter muscle thickness is correlated to vertical facial pattern, showing that individuals with thicker masseter have a vertically shorter face [1,2]. Conversely, Farella et al. [3] found that the daily long-term activity of masseter muscle seems to be similar in short-face and long-face subjects in the natural environment. A recent review on mandibular muscles and vertical facial pattern highlighted that there is no conclusive evidence of the influence of mandibular muscles on normal growth and development of the face [4]. Thus, it still is unknown whether craniofacial morphology, or pattern, has an impact on function, and whether muscular function affects facial geometry.

Maximum occlusal force (MOF) may be considered a measure of masticatory muscles function because represents the effort exerted between the maxillary and mandibular teeth when the mandible is elevated. The large intersubject variability of MOF results from a complex interaction of many factors such as sex, age, body mass index, presence of temporomandibular disorders, size and direction of the masseter muscle, craniofacial morphology, dental occlusal status, periodontal sensitivity, and psychological factors [5-8]. Raadsheer et al. [6] stated that the magnitude of MOF depends on the size of the jaw muscles and the lever arm lengths of MOF and muscle forces, which would be related to craniofacial morphology.

Regarding mandibular elastic deformation, medial convergence, corporal rotation and dorsoventral shear occur simultaneously during functional movements and are related to muscular closing forces and jaw position [9]. Medial mandibular flexure (MMF) is a mandibular deformation characterized by a decrease in arch width during jaw opening and protrusion movements because of the functional contraction of the lateral pterygoid muscles, causing high strain in the symphyseal region [10-12]. Therefore, it would be reasonable to expect that stronger muscles would be associated with larger mandibular flexure. The influence of geometric facial factors on mandibular deformation is unclear as only a few measures have been found to be statistically significant. For example, some in vivo studies observed that the highest values of mandibular deformation occurred in subjects with lower symphysis height [9,12]. Also, Chen et al. [13] found that subjects with larger mandibular length, lower gonial angle and smaller symphysis area had the highest mandibular deformation. There is a lack of data from Latin American populations, and no other indexed paper evaluated both occlusal force and mandibular deformation in relation to vertical facial pattern.

This study reports data analysis from a subsample of a previous work, where we tested the association between MOF and MMF [14]. No statistically significant correlation was then found between MOF and MMF in maximum opening or in maximum protrusion in a sample composed by 80 dentate adults. Cephalometric data were not available for the sample by that time though. Therefore, this study tested whether the variation in vertical facial pattern affects voluntary MOF and MMF in a sample of fully-dentate Brazilian adults. The a priori hypothesis was that MOF and MMF vary as a function of vertical facial pattern.

## Methods

The research project of this cross-sectional study was approved by the University's Ethics Committee (SISNEP CAAE-0094.0.002.000-02) in compliance with the Helsinki Declaration, and all participants signed an informed consent form. A convenience sample was recruited from the students and faculty of the Pontifical Catholic University of Rio Grande do Sul Dental School. Eligibility criteria comprised complete dentition (facultative presence of third molars), age range from 20 to 50 years old, and normal occlusion. Exclusion criteria were: history of maxillofacial surgery, mandibular trauma or orthodontic treatment within the previous two years; presence of active periodontal disease with tooth mobility, osseous or neuromuscular diseases, marked jaw asymetries, orofacial pain, or pregnancy.

### Anthropometric measures

Subject's height was measured in centimeters (cm) with the subject in erect position without shoes, and the weight was recorded in kilograms (kg) using a mechanical anthropometric scale (Welmy, model R110, Santa Bárbara do Oeste, SP, Brazil). The body mass index (BMI) was computed using the formula: BMI = weight/height^2 ^(kg/m^2^).

### Vertical facial pattern

A lateral cephalometric radiograph of each subject was taken following a standardized protocol with the teeth in occlusion. The cephalometric tracings and analyses were performed manually by a certified orthodontist (FLL) according to Ricketts et al. [15]. The vertical facial pattern was determined by computing the VERT index [15] using five mandibular measurements (mandibular plan, facial axis, anterior lower facial height, mandibular arch, and facial depth) and the normative values according the subject's age. The VERT index is the arithmetic mean of the difference between the five cephalometric measures and the values considered ideal for a harmonic face, divided by the standard deviation. The signal is negative when the growth trend is vertical, and positive when is horizontal. The facial pattern of each subject was classified as: Dolichofacial (below -0.5), Mesofacial (between -0.49 and +0.49), and Brachyfacial (above +0.5).

### Maximum Occlusal Force (MOF)

A compressive load transducer (Sensotec13/2445-02, Columbus, OH, USA) was used to measure MOF in the first molar region. The bite pad containing the load transducer was covered with a hard rubber band, and the set was wrapped with disposable plastic film. The interocclusal distance of the bite pad at the insertion point was 14 mm. After instructions and training, the subject was asked to bite the equipment five times with maximal effort for 1 to 2 seconds, with rest intervals between trials. The three highest measures were averaged and considered the subject's MOF value (in newtons) [7].

### Medial Mandibular Flexure (MMF)

MMF was measured by calculating the variation of the intermolar distance from rest (R) to maximum opening (O) positions using an impression technique [14]. For each subject, impressions of the occlusal and incisal thirds of the mandibular teeth were obtained during relative rest (minimum mouth opening for impression making) and maximum opening using a vinyl polysiloxane putty material (3 M Express, Saint Paul, MN, USA).

The impressions and digital calipers with the measuring head set at a 10 mm-width were scanned at 200% magnification and 300 dpi resolution. Using the Adobe Photoshop^® ^4.0 software tools, anatomical reference points on the contralateral first molars were selected for the images (R, O). The intermolar linear distance was measured with the Image Tool software (University of Texas Health Science Center at San Antonio, San Antonio, TX, USA) [16]. Before the intermolar measurement, each image was calibrated with the digital calipers image (10 mm-width). Intermolar distance was measured in triplicate for each image and averaged.

MMF in maximum opening was calculated by subtracting the intermolar distance at O position from R position. A single calibrated examiner, who was blind to occlusal force values and cephalometric data, performed all linear measurements and MMF calculations. Reliability tests of this method yielded intra-rater intraclass correlation coefficients (ICC) from 0.98 to 0.99, inter-rater ICC of 0.69 [17], and test-retest ICCs from 0.96 to 0.99 [14].

### Statistical analysis

The main outcome measures were MMF (in millimeter) and MOF (in newton), which presented normal distribution and homogeneity of variances. Data were analysed by analysis of covariance (ANCOVA) at the 0.05 level of significance. The fixed factors were Vertical facial pattern (dolichofacial, mesofacial, brachyfacial) and Sex (males, females); the covariate was BMI (in kg/m2). The residues generated after the application of the statistical model followed normal distribution. All statistical tests were two-tailed, and a P-value of 0.05 was considered statistically significant for rejection of the null hypothesis.

## Results

Cephalometric data were available for 51 subjects. Descriptive characteristics of this sample are shown in Table 1. In relation to the frequency of facial types, there was a predominance of brachycephalic types.

**Table 1:** Sample demographic and clinical characteristics (n = 51).

**VARIABLE**	**FREQUENCY (%)**	**MEAN (SD)**	**MINIMUM – MAXIMUM**
SEX			
Females	27 (53)		
Males	24 (47)		
FACIAL PATTERN			
Brachyfacial	35 (68.6)		
Females	18		
Males	17		
Mesofacial	10 (19.6)		
Females	6		
Males	4		
Dolychofacial	6 (11.8)		
Females	3		
Males	3		
VERT INDEX		1.07 (1.24)	-1.70 – 3.50
Brachyfacial		1.73 (0.79)	0.50 – 3.50
Mesofacial		0.09 (0.31)	-0.40 – 0.40
Dolychofacial		-1.13 (0.38)	-1.70 – -0.60
AGE (years)		24.6 (3.9)	20 – 37
Brachyfacial		24.5 (3.7)	20 – 34
Mesofacial		23.1 (1.9)	20 – 26
Dolychofacial		27.3 (6.4)	21 – 37
BMI (kg/m^2^)		22.0 (2.5)	16.7 – 28.4
Brachyfacial		22.1 (2.9)	16.7 – 28.4
Mesofacial		21.6 (1.2)	20.3 – 24.1
Dolychofacial		21.8 (2.1)	19.1 – 25.1

No significant difference of MOF or MMF was found among the three facial patterns (P = 0.62 and P = 0.72, respectively) (Table 2). BMI was not a significant covariate for MOF or MMF (P > 0.05). Sex was a significant factor only for MOF (P = 0.007); males had higher MOF values than females.

**Table 2:** Maximum occlusal force (MOF) and medial mandibular flexure (MMF) as a function of facial pattern.

	**VERTICAL FACIAL PATTERN**			
		
**OUTCOME VARIABLE**	**Dolychofacial** **(n = 6)** Mean (SD)	**Mesofacial** **(n = 10)** Mean (SD)	**Brachyfacial** **(n = 35)** Mean (SD)	**TOTAL** **(n = 51)** Mean (SD) *
MOF (N)				
Males	843 (256)	1014 (426)	1046 (265)	1003 (287) a
Females	642 (215)	705 (158)	654 (190)	664 (180) b
Total	742 (238)	829 (316)	831 (295)	820 (289)
MMF (mm)				
Males	0.07 (0.07)	0.25 (0.21)	0.15 (0.16)	0.16 (0.16)
Females	0.22 (0.11)	0.19 (0.28)	0.20 (0.21)	0.20 (0.21)
Total	0.15 (0.11)	0.21 (0.25)	0.18 (0.18)	0.18 (0.19)

*Means followed by distinct letters are statistically different at α = 0.05

## Discussion

Previous studies showed that greater hyperdivergence is related to poorer mechanical advantage and lower maximum bite force in children [18] and adults [6]. Because of the potential differences of muscular force vectors in different facial patterns [4], it was expected that brachyfacial subjects had higher muscular force than dolichofacial subjects. Contrary to our hypothesis, we did not find any significant effect of vertical facial pattern on either MOF or MMF. The post hoc power analysis for a total of 51 cases, alpha = 0.05, yielded a power of 9% for MOF (calculated effect size: 0.10) and 8% for MMF (calculated effect size: 0.09) (Cohen's conventions for F test: small = 0.10, medium = 0.25, large = 0.40). To increase power to 80% and considering the same effect sizes, the required total sample sizes would be 933 subjects to detect differences in MOF and 1134 subjects for MMF. However such small effects might be clinically irrelevant and not worth recruiting the large numbers of subjects needed to show statistical significance. If there was a medium sized effect (0.25) our study would have about 88% power at alpha = 0.05. So if a medium or large effect with clinical relevance did exist in the target population, then our sample size should be large enough to detect it. A limitation of our study was the unequal allocation of facial patterns. Brazilian population reflects an ethnoracial mixture of immigrants from many parts of the world and several local Indian peoples with diverse genetics. Our study sample was drawn from a convenience sample of dental students and staff in the South region of Brazil, where dolichofacial and mesofacial patterns are less frequent than the brachyfacial type.

The negative results of the effect of facial pattern on MOF in our sample of Brazilian dentate adults (20–38 years-old) are supported by other studies conducted in different populations. Kiliaridis et al. [19] found that maximum bite force in the molar area was not associated with facial characteristics in Scandinavian children and young adults, although they found that bite force in the incisor region was higher in short lower anterior height. Tuxen et al. [20] also reported no significant association between bite force and facial morphology among men and women subsamples.

In relation to the variables adjusted for in our analyses, BMI was not a significant covariate for MOF or MMF (P = 0.43 and P = 0.48, respectively). Our sample was quite homogeneous for BMI (mean: 21.9, SD: 2.4), but we included this variable as a covariate in our models because some studies showed association of occlusal force with weight and height. Sex was a significant factor only for MOF, and males had higher MOF values than females. These results parallel previous reports, which partially explained the larger bite force in males by the larger diameter and cross-sectional area of type II fibres of the males' masseter muscle compared to the females' counterpart [20].

MOF represents the sum of forces exerted by the stomatognathic system during maximum occlusion of teeth, and multiple factors are involved. Hatch et al. [7] tested a multivariate model of masticatory performance and found that the combined effects of sex, number of functional posterior tooth units, masseter cross-sectional area, age, and presence of temporomandibular disorders explained 52% of the variance of bite force in adult European- and Mexican-Americans. Bite force was influenced primarily by sex and number of functional tooth units. Age and sex were significant determinants of muscle cross-sectional area, but the association between sex and masseter cross-sectional area was not strong enough to explain the sex-related variation in bite force. In another multivariate approach, 58% of the variance of bite force magnitude in adults was explained by craniofacial morphology and masseter muscle thickness [6]. Bite force magnitude had a positive association with thickness of the masseter muscle, vertical and transverse facial dimensions and the inclination of the midface, and negative association with mandibular inclination and occlusal plane inclination. Radsheer et al. [6] also found that the contribution of the masseter muscle to the variation in bite force magnitude and moment was higher than that of the craniofacial factors.

Regarding the muscles per se, there is a large inter-subject variability of size and shape of muscular attachments, jaw muscle insertions alter position during functional movements, and their displacement patterns vary according to the muscle [21]. Also, Goto et al. [22] showed that the deep and superficial regions of the masseter muscle do not stretch uniformly during major jaw movements. For instance, on maximum opening, the medial part of the deep masseter showed the largest increase in muscle length, and the smallest changes occurred in the posterior-most, superficial masseter. Each muscle part moves differently according to variations in the size and shape of insertion areas, musculoskeletal form, and patterns of jaw motion during function [21].

Similarly to occlusal force, there was no effect of vertical facial pattern on mandibular elastic deformation. Previous studies showed a positive bivariate association of mandibular deformation with lower symphysis height smaller symphysis area, larger mandibular length, and lower gonial angle [13]. However, it may be more difficult to clinically predict mandibular deformation on a basis of isolated facial measures instead of a summary outcome measure, such as facial pattern. For example, Gesch et al. [23] found that bivariate and multivariate analysis methods lead to different results when the craniofacial pattern of Class II/1 malocclusion subjects was compared with normal occlusion or Class I malocclusion subjects. Also, Throckmorton et al. [24] tested a factor analysis of multivariate sagittal and biomechanical factors of craniofacial morphology and found that only measurements of relative differences between anterior and posterior facial height were strongly correlated with maximum bite force. We classified the vertical facial pattern using the VERT index [15], which is a composite of cephalometric measures, namely the mandibular plan, facial axis, anterior lower facial height, mandibular arch, and facial depth. As a result, we considered that the use of vertical facial pattern in the analyses of MOF and MMF would provide a more global and interactive measure of craniofacial morphology.

## Conclusion

Our results do not support that MOF and MMF vary as a function of vertical facial pattern in this sample of Brazilian dentate adults. Thus, functional outcome measures represented by muscular force and mandibular flexure would be a result of far more contributing factors than craniofacial morphology and other variables assessed here.

## Competing interests

The author(s) declare that they have no competing interests.

## Authors' contributions

RSS conceived the study design, performed the data analysis and wrote the manuscript. FLL, SAC, and MG performed the subjects' enrollment, data collection and data analysis. MLG participated in the early preparation of the manuscript. LMH and EGM contributed to write the revised version of the article. All authors read and approved the final version of the manuscript.
